# Genome-wide association analyses of North American Rheumatoid Arthritis Consortium and Framingham Heart Study data utilizing genome-wide linkage results

**DOI:** 10.1186/1753-6561-3-s7-s103

**Published:** 2009-12-15

**Authors:** Yun Joo Yoo, Dushanthi Pinnaduwage, Daryl Waggott, Shelley B Bull, Lei Sun

**Affiliations:** 1 Samuel Lunenfeld Research Institute of Mount Sinai Hospital, 600 University Avenue, Toronto, Ontario M5G 1X5 Canada; 2 Dalla Lana School of Public Health, University of Toronto, 6th Floor, Health Sciences Building, 155 College Street, Toronto, Ontario M5T 3M7 Canada; 3 Department of Statistics, University of Toronto, 100 St. George Street, Toronto, Ontario M5S 3G3 Canada

## Abstract

The power of genome-wide association studies can be improved by incorporating information from previous study findings, for example, results of genome-wide linkage analyses. Weighted false-discovery rate (FDR) control can incorporate genome-wide linkage scan results into the analysis of genome-wide association data by assigning single-nucleotide polymorphism (SNP) specific weights. Stratified FDR control can also be applied by stratifying the SNPs into high and low linkage strata. We applied these two FDR control methods to the data of North American Rheumatoid Arthritis Consortium (NARAC) study and the Framingham Heart Study (FHS), combining both association and linkage analysis results. For the NARAC study, we used linkage results from a previous genome scan of rheumatoid arthritis (RA) phenotype. For the FHS study, we obtained genome-wide linkage scores from the same 550 k SNP data used for the association analyses of three lipids phenotypes (HDL, LDL, TG). We confirmed some genes previously reported for association with RA and lipid phenotypes. Stratified and weighted FDR methods appear to give improved ranks to some of the replicated SNPs for the RA data, suggesting linkage scan results could provide useful information to improve genome-wide association studies.

## Background

Use of prior or additional information may improve the power of single-nucleotide polymorphism (SNP)-disease association analysis. In particular, genome-wide linkage scans can provide complementary information to genome-wide association studies (GWAS). The weighted false-discovery rate control (WFDR) method incorporates genome-wide linkage study results by converting the linkage scores into SNP-specific weights then re-scaling the association *p*-value for each SNP [1]. The stratified FDR control method (SFDR [2]) prioritizes genomic regions according to the available linkage scores. WFDR requires the investigator to choose and assign weights to SNPs whereas in SFDR, stratum-specific weights are internally derived by the choice of strata and the distribution of data [3]. SFDR is designed to use prior information to assign SNPs into strata that are more or less likely to include true-positive associations, which can similarly improve the power of GWAS, but is more robust than WFDR to uninformative or even misleading prior information [3]. We applied these two FDR methods along with the original FDR method to the North American Rheumatoid Arthritis Consortium (NARAC) study data provided for Genetic Analysis Workshop 16 (GAW 16) using previously reported linkage study results for rheumatoid arthritis (RA) [4]. We also performed genome-wide linkage and association analyses of the FHS data and applied the three FDR methods. We compared the regions of association identified by the different methods.

## Methods

### Samples, phenotypes, and genotypes

#### RA data

The NARAC data provided for GAW 16 included a set of 868 cases and 1,194 controls with information on a binary outcome (RA affection status) and on 545,080 genome-wide SNP genotypes from the Illumina 550 k chip as well as the physical locations for the SNPs. In our association analysis, RA affection status was defined as positive for anti-cyclic citrinullated peptide antibody (anti-CCP).

#### FHS data

SNP genotyping data were provided based on the GeneChip Human Mapping 500 k Array and 50 k Human Gene Focused Panel. We analyzed a combined sample of the Offspring Cohort (*N*_1 _= 2,584) and the Generation 3 Cohort (*N*_2 _= 3,811) for association with each of the blood lipid measures, high-density lipoprotein (HDL), low-density lipoprotein (LDL), and triglyceride (TG), and included all family members in the sample who had been genotyped and phenotyped. The mean of lipid measures over multiple exams was adjusted for age, sex, body mass index (BMI), alcohol intake, and cigarette smoking. The phenotype measures of treated people were imputed using the methods in Kathiresan et al. [5] and Levy et al. [6].

### Quality control of SNP data

We excluded SNPs with Hardy-Weinberg equilibrium *p*-value ≤ 10^-9^in controls, missing genotype rate >5%, and minor allele frequency <0.01. Samples were also filtered by individual call missing rate >5%, duplicity, and relatedness. Within autosomes, there were 490,915 SNPs remaining in the RA data and 430,292 SNPs in the FHS data after applying this set of quality control criteria, using the computer program PLINK [7].

### Genome-wide association analysis

#### RA data

Each SNP was tested for association using the 1-df allelic chi-squared test assuming an additive genotype model, implemented in PLINK [7].

#### FHS data

SNP association was evaluated using adjusted residual mean values obtained from the generalized estimating equation model for familial correlation. We split families unconnected in the Offspring and Generation 3 Cohort using the R package "kinship" [8]. Generalized estimating equation fitting was performed using a SAS GENMOD procedure assuming an exchangeable working correlation matrix.

### Genome-wide linkage analysis

#### RA data

Results of the NARAC linkage study of RA using 642 Caucasian families and high-density SNP genotyping, as reported in Amos et al. [4], were used as the available prior linkage information, based on RA status (anti-CCP positivity) as the phenotype. The linkage scores at SNP markers across the genome, publicly available as supplementary information, were the nonparametric linkage (NPL) scores computed by Amos et al. [4] using linkage disequilibrium (LD) eliminated SNP genotypes. For the chromosomes with large centromeres (1, 3, 9, 11, 16, and 19), they assumed zero recombination of the centromeric regions.

#### FHS data

We performed a genome-wide linkage scan for each of the three lipid phenotypes values (i.e., covariate adjusted residuals), using 8,545 individuals from 1,349 FHS families (3,928 founders, 4,617 non-founders; 4,363 females and 4,182 males; family size ranging from 3 to 19). We selected 5,102 SNPs for the linkage scan according to the criteria of MAF > 0.45, HWE test *p*-values in founders >0.05, individual genotype missing rate <5%, SNP missing rate <2%, mendelian error rate <5%, and LD measure *r*^2 ^< 0.05 between SNPs. Genome-wide linkage scans were performed using the regression methods of MERLIN-REGRESS (version 1.1.2) [9,10]: the identical-by-descent (IBD) allele-sharing status for all relative pairs was regressed on the squared differences and squared sums of the pairs' trait values. This method requires specification of the population trait mean, variance, and heritability. We therefore estimated the heritability using the variance-components (VC) option in MERLIN. Lacking an available genetic map, we interpolated the deCODE map from the Affymetrix, Inc. website for the 5,102 selected SNPs.

#### SNP-specific linkage scores

The linkage score corresponding to each of the ~550 k GWA SNPs, *Z*_*i*_, *i *= 1, ..., *M*, was interpolated from the linkage scores of the available neighboring markers according to the relative distance between markers.

### False-discovery rate control methods

False-discovery rate (FDR) control was performed by computing *q*-values using the method suggested by Storey [11]. If the *q*-value of a single SNP analysis was less than the chosen FDR control threshold value, the hypothesis of no association between the SNP and the disease was rejected.

#### Stratified FDR

Based on the linkage scan results, high and low linkage regions were determined using a NPL threshold value of *C *= 1.64 for the NARAC RA data and LOD threshold value *C *= 0.5 for the FHS data. SNPs that fell into a high-linkage region were grouped as Stratum 1 (*Z*_*i *_≥ *C*) and SNPs that fell into a low-linkage region were grouped as Stratum 2 (*Z*_*i *_<*C*). FDR control was applied separately for the SNPs in Stratum 1 and Stratum 2 [11].

#### Weighted FDR

The weight of each SNP was obtained as *w*_*i *_= exp(*B·Z*_*i*_)/*v*, where  and *B *= 1 (exponential weighting [1]). FDR was applied to weighted *p*-values, *p*_*j*_/*w*_*j*_, and the corresponding *q*-values were computed.

## Results

### Results of the RA data analysis

The SNPs in chromosome 6 (MHC region) showed very strong association with *p*-values less than 10^-100 ^and also very high linkage scores (NPL>16). To focus on results outside regions of established importance, Table 1 excludes chromosome 6 SNPs and presents results of SNPs with ranks ≤ 10 based on any of FDR methods or SNPs from genes previously reported to be associated with RA [12-14]. Most of the latter were ranked higher than other SNPs in the genome. For some SNPs, mostly in the stronger linkage regions, either SFDR or WFDR improved the rank more than the other. For example, the original rank of rs1018361 in *CTLA4 *was 285 using FDR, which changed to 28 and 96 using SFDR and WFDR, respectively. In some weak linkage regions (*TRAF1*, *WDFY4*), WFDR retained similar ranks as FDR, whereas the SFDR ranks increased. However, *q*-values for WFDR were generally much higher than those of FDR and SFDR (results not shown). These analyses suggest several new associations with RA (e.g., *CNTNAP2 *on chromosome 7).

**Table 1:** Results of FDR, SFDR, and WFDR analyses of selected SNPs for the RA phenotype from the NARAC study

SNP^a^	Chr	BP	Gene	Association *p*-value	Rank			Linkage NPL score^c^
						
					FDR	SFDR	WFDR	
rs6683201	1	17426583	*PADI4*^b^	1.32 × 10^-3^	3531	4172	5320	-0.11
rs2476601	1	114089610	*PTPN22*^b^	2.91 × 10^-12^	1	1	1	0.1
rs2542941	2	172235228	*CYBRD1*	4.25 × 10^-6^	69	5	36	**1.76**
rs6433309	2	172343658	No gene	1.50 × 10^-6^	35	2	21	**1.74**
rs13031008	2	178302621	*TTC30A*	8.13 × 10^-6^	121	11	38	**2.24**
rs12693591	2	191686008	*STAT1*^b^	3.98 × 10^-2^	39083	15997	5028	**3.38**
rs6752770	2	191799069	*STAT4v*	7.28 × 10^-3^	11404	2787	1554	**3.42**
rs10184573	2	200273759	No gene	5.63 × 10^-6^	84	6	22	**2.93**
rs1018361	2	204510341	*CTLA4*^b^	3.36 × 10^-5^	288	28	98	**2.14**
rs6596147	5	133075674	No gene	4.61 × 10^-9^	3	8	4	0.16
rs6978820	7	146629802	*CNTNAP2*	2.22 × 10^-6^	46	3	24	**1.79**
rs2900180	9	120785936	*TRAF1*^b^	3.08 × 10^-9^	2	7	3	0.01
rs7037673	9	120820038	*C5*^b^	2.15 × 10^-5^	212	400	306	0.01
rs2671692	10	49767825	*WDFY4*	2.66 × 10^-8^	10	18	10	0.28
rs1182531	20	57826397	*PHACTR3*	4.83 × 10^-9^	4	9	2	0.58
rs9974986	21	35262686	*RUNX1*^b^	3.79 × 10^-3^	7246	8165	6401	0.68
rs713756	22	43118847	No gene	2.10 × 10^-8^	9	17	8	0.08

^a^The SNPs listed in the table include the most significant SNP from each of the previously reported genes and SNPs with ranks ≤ 10 (one SNP per region) based on any of the FDR methods.

^b^Genes previously reported to be associated with RA.

^c^Genome-wide linkage NPL scores ≥ 1.64 (the chosen SFDR threshold) are in bold.

### Results of the FHS data analysis

Table 2 presents the results of SNPs selected with ranks ≤ 10 based on any of the three FDR methods or those most significant among genes previously reported for association with TG [5,15]. All SNPs with rank ≤ 10 resided in the previously reported genes. SNPs in stronger linkage regions showed improvement in rank using SFDR and WFDR (linkage scores in bold). However, some of the gene regions previously reported for TG, HDL, or LDL were not confirmed in the FHS samples.

**Table 2:** Results of FDR, SFDR, and WFDR analysis of selected SNPs for the TG phenotype from the FHS study

SNP^a^	Chr	BP	Gene^b^	Association *p*-value	Rank			Linkage LOD score^c^
						
					FDR	SFDR	WFDR	
rs4350231	1	62695248	*DOCK7*	2.39 × 10^-3^	2376	2953	2755	0.11
rs4846918	1	228367209	*GALNT2*	2.00 × 10^-2^	14171	9915	10990	**0.56**
rs780094	2	27594741	*GCKR*	2.83 × 10^-10^	18	16	16	**1.1**
rs6731583	2	202269099	*ALS2*	9.12 × 10^-2^	52483	55094	66274	0
rs16872759	4	22118656	*GPR125*	6.90 × 10^-3^	5805	6490	6951	0.05
rs1178977	7	72494985	*BAZ1B*	2.17 × 10^-12^	10	12	10	0
rs17145738	7	72620810	*TBL2*	1.28 × 10^-10^	15	19	18	0
rs17411031	8	19896590	*LPL*	1.23 × 10^-17^	1	1	2	**0.91**
rs2980875	8	126550929	*TRIB1*	1.74 × 10^-9^	21	24	21	0.38
rs6475522	9	2120917	*SMARCA2*	1.62 × 10^-2^	11891	7794	5774	**1.14**
rs3750929	11	82259235	*PRCP*	1.73 × 10^-1^	92300	96678	115272	0
rs6589566	11	116157633	*APOA5*	1.71 × 10^-12^	9	11	9	0.02
rs948028	11	120149657	*GRIK4*	1.80 × 10^-3^	1899	2234	1986	0.23
rs2451214	17	78308343	*TBCD*	1.98 × 10^-1^	103764	108501	129319	0
rs3813136	19	15452333	*PGLYRP2*	7.23 × 10^-1^	333987	276706	250668	**0.51**
rs2424295	20	20165327	*C20orf26*	2.30 × 10^-2^	15945	17260	18677	0.09

^a^The SNPs listed in the table include the most significant SNP from each of the previously reported genes and SNPs with ranks ≤ 10 (one SNP per region) based on any of the FDR methods.

^b^All genes listed were previously reported to be associated with RA.

^c^Genome-wide linkage LOD scores ≥ 0.5 (the chosen SFDR threshold) are in bold.

## Discussion

The SFDR and WFDR methods can improve power of genome-wide association analyses when linkage scans are informative [1,3]. In the RA and FHS studies, using SFDR and WFDR improved ranks of SNPs in new and previously reported regions, suggesting improved power.

The threshold value for Stratum 1 and 2 in SFDR was somewhat arbitrarily set as 1.64 for the RA data (where the linkage results were NPL scores) and 0.5 for the FHS data (where the linkage results are LOD scores) to maintain the proportion of Stratum 1 to be about 5% when the power of linkage scans is relatively low. The effect of small differences in threshold values for SFDR was insignificant on average in a simulation study [3]. In the RA and FHS data sets, the choice of different thresholds did produce some differences in ranking values, but the effect was minimal.

The power under FDR control depends on the proportion of true alternative hypotheses (true causal SNPs) in a family of tests. By preserving most of the potentially true causal SNPs in a small-sized Stratum 1, we can improve study power [3]. However, how to choose this threshold to optimize power remains an open question. A similar question applies to the choice of the weighting scheme (i.e., the value of the *B *parameter) for the WFDR method.

SFDR and WFDR control methods using previous linkage results can be extended to multi-marker analysis with fixed or sliding windows, for example, by using the average linkage score within a window as a measure of prior information. Further study is warranted to evaluate extensions to multi-marker analysis settings.

## List of abbreviations used

Anti-CCP: Anti-cyclic citrinullated peptide antibody; BMI: Body mass index; FDR: False-discovery rate; GAW: Genetic Analysis Workshop; GWAS: Genome-wide association studies; HDL: High-density lipoprotein; IBD: Identical by descent; LD: Linkage disequilibrium; LDL: Low-density lipoprotein; NARAC: North American Rheumatoid Arthritis Consortium; NPL: Nonparametric linkage; RA: Rheumatoid arthritis; SFDR: Stratified false-discovery rate control method; SNP: Single-nucleotide polymorphism; TG: Triglyceride; VC: Variance-component; WFDR: Weighted false-discovery rate control.

## Competing interests

The authors declare that they have no competing interests.

## Authors' contributions

YJY contributed to the design and writing of the paper; association analysis of FHS data; and SFDR and WFDR analysis of RA and FHS data. DP contributed to the design and writing of the paper, association analysis of RA, data and linkage analysis of FHS data. DW contributed association analysis of FHS data and QC analysis of RA and FHS data. SBB contributed to the critical revision of the paper for important content. LS contributed to the design of the paper and critical revision of the paper for important content.
